# Training items for trainee residents in community medicine in rural areas

**DOI:** 10.1186/s13104-023-06594-7

**Published:** 2023-10-30

**Authors:** Ayako Kumabe, Tsuneaki Kenzaka, Naoya Mizutani, Ken Goda, Shinsuke Yahata

**Affiliations:** 1 Division of Community Medicine and Career Development, Kobe University Graduate School of Medicine, 2-1-5, Arata-cho, Hyogo-ku, Kobe, 652-0032 Hyogo Japan; 2 Department of Internal Medicine, Hyogo Prefectural Tamba Medical Center, 2002-7 Iso, Hikami-cho, Tamba, 669-3495 Japan; 3 Division of Community Medicine and Medical Education, Kobe University Graduate School of Medicine, 2-1-5 Arata-cho, Hyogo-ku, Kobe, 652-0032 Japan; 4 Department of General Internal Medicine, Hyogo Prefectural Harima-Himeji General Medical Center, 3-264 Kamiya-cho, Himeji, 670-0836 Japan

**Keywords:** Significant event analysis, Resident training, Community medicine, Rural medicine, Trainee residents

## Abstract

**Objective:**

To examine the significant events experienced by initial trainees during community medicine training, evaluate their impact on community medicine practice, and support improvements in rural community medicine training.

**Results:**

Three faculty teachers independently evaluated the reports of 25 residents who had completed a four-week community medicine training in a rural area to analyze major events. The reports were analyzed using topics from the Model Core Curriculum for Medical Education that relate to rural medicine. The most frequently reported items were identified as follows: Primary care: 9 (36.0%); integrated community care systems: 8 (32.0%); medical care in the local community: 7 (28.0%); home health care and systems, patient-physician relationship, and end-of-life medical treatment and care: 6 each (24.0%). Reports from residents describing events related to home health care and systems and end-of-life medical treatment and care were related to more than one item.

## Introduction

Community-based medical education (CBME) is essential to develop a holistic patient care mindset and improve the quality of care [1]. CBME is also a practical educational method for residents to learn about primary care and family medicine at institutions other than universities and tertiary hospitals [2, 3].

Since 2004, in Japan, clinical residents must complete initial clinical residency training within two years of medical school [4]. This program includes four weeks of community medicine mandatory training during the second post-graduate year [4]. The objectives of community medicine training outlined by Japan’s Ministry of Health, Labour and Welfare are aimed to instill in residents the ability to “understand the characteristics of regional medical care as well as the concept and framework of community medicine while also cooperating with a variety of facilities and organizations related to medical care, long-term care, healthcare, and welfare [4].” These guidelines state that training sites should be either clinics or hospitals located in rural areas or islands that have less than 200 beds. Although these guidelines feature the requirements for community medicine training sites, no specific training content is provided [4]. Although these guidelines specify the location for training, they do not outline a specific methodology for achieving the comprehensive patient care attitude in CBME [1–4].

In this study, we employed a significant event analysis (SEA) framework to facilitate reflection by trainee residents on their community medicine training [5]. Our method included a systematic and detailed review of significant cases for individual physicians (independent of whether undesirable patient outcomes occurred) to improve the quality of future practice. The SEA procedure consists of the following six steps: (1) Describe the significant event; (2) Describe the practitioner’s initial thoughts and feelings at the time; (3) Detail what went well; (4) Detail what did not go well; (5) Describe what could have been done better; (6) Outline action and learning plans for the future [6].

Clinical residency training is primarily conducted in hospitals with 200 or more beds that function as large hospitals. We believe that the content of community medicine residency training should focus on lessons that can only be learned in the community medicine setting and that are distinctly different from those often learned in large hospitals.

Furthermore, the significant events experienced by resident trainees during community medicine training can serve as critical training items to inform future community medicine training, thus being the foundation of CBME. Thus, the purpose of this study was to identify and examine the content of SEA reports by resident trainees in community medicine settings in Japan to inform the development of future training programs.

## Materials and methods

### Study design

This was a retrospective descriptive study using data from SEA reports completed by resident trainees after four weeks of community medicine training. This study was conducted in accordance with the Declaration of Helsinki. Ethical review was deemed unnecessary for this study by the ethics committee of Kobe University Hospital Clinical and Translational Research Center, as we used existing anonymized resident training reports with blanket permission for academic. Written consent for academic use was obtained from residents.

### Community Medicine Training settings

In Japan’s two-year initial clinical residency training system, four weeks of clinical training in community medicine is mandatory in the second academic year. Kobe University’s community medicine training program comprises three sub-programs: community medicine training at urban clinics, community medicine training at mid-size hospitals in rural areas with less than 200 beds, and community medicine training at small hospitals in rural areas. Residents who have completed their training in community medicine in a small hospital in a rural area must complete a four-week SEA reflection at the end of their training and submit a report for the Kobe.

### Participants

All 25 residents who received community medicine training at the Kobe University Graduate School of Medicine participated in this study. Trainees completed rural training in 2017 and 2018 and completed reports after four weeks SEA collected for analysis.

### Data analysis

Report Analysis Procedure.

Referring to the content related to community medical training in the Model Core Curriculum for Medical Education [7], training items were classified into the following 10 categories:

(1) Medical care in the local community, (2) Medical planning and regional medical care (including cooperation between hospitals and diagnostic services), (3) Integrated community care systems (interdisciplinary cooperation), (4) Role of primary care, (5) Emergency medical care and systems, (6) Home health care and systems, (7) Disaster medicine services and readiness, (8) Patient–physician relationship, (9) Team medicine, (10) End-of-life medical treatment and care.

Three faculty members independently assessed the content of SEA reports prepared by residents who had completed a four-week community medicine training in a rural area. The content of the reports was examined and distinguished according to the ten items listed above. Content was assigned to the most relevant item, and content that applied to more than one item was considered to apply to each relevant item. In cases where teachers’ evaluations were inconsistent, agreement of the applicable item was determined through discussion.

## Results

All participants (20 males and five females) were physicians in their second year of post-graduate training. The following items were identified in the SEA reports most frequently: (4) role of primary care: 9 (36.0%); (3) integrated community care systems: 8 (32.0%); (1) medical care in the local community: 7 (28.0%); (6) home health care and systems; (8) patient–physician relationship; (10) end-of-life medical treatment and care: 6 (24.0%) each. Data for all items in the SEA reports are presented in Table 1.

**Table 1:** Frequency of applicable items from SEA reports

Training items	Number of reports (%)
(4) Primary care	9 (36.0%)
(3) Integrated community care system	8 (32.0%)
(1) Medical care in the local community	7 (28.0%)
(6) Home health care and systems	6 (24.0%)
(8) Patient–physician relationship	6 (24.0%)
(10) End-of-life medical treatment and care	6 (24.0%)
(5) Emergency medical care and systems	5 (20.0%)
(9) Team medicine	5 (20.0%)
(2) Medical planning and regional medical care	3 (12.0%)
(7) Disaster medicine	0 (0.0%)

Table 2 shows which items were derived from each SEA report. Only five reports contained one item per resident, with most residents reporting multiple items. In particular, the reports of residents who experienced significant events related to (6) home health care and systems and (10) end-of-life medical treatment and care applied to more than one item.

**Table 2:** Applicable items for each training report

Resident report number	(4) Primary care	(3) Integrated community care systems	(1) Medical care in the local community	(6) Home health care and systems	(8) Patient–physician relationship	(10) End-of-life medical treatment and care	(5) Emergency medical care and systems	(9) Team medicine	(2) Medical planning and regional medical care	(7) Disaster medicine
1	+					+		+		
2	+				+					
3	+									
4	+									
5	+						+			
6	+				+					
7	+				+				+	
8	+									
9	+	+		+	+					
10		+	+	+						
11		+	+							
12		+		+	+	+				
13		+			+					
14		+								
15		+	+	+						
16		+				+		+		
17			+			+				
18			+				+		+	
19			+							
20			+	+						
21				+		+		+		
22						+		+		
23							+	+	+	
24							+			
25							+			
total	9	8	7	6	6	6	5	5	3	0

+ indicates that events recorded in the report pertained to the item

Overall, the average number of items per report was 2.2. The average number of (6) home health care and systems is 3.6 items per report and (10) end-of-life medical treatment and care is 2.8 items per report fell into this category.

## Discussion

In their SEA-style reports, residents frequently reported significant events related to primary care and integrated community care systems. It is difficult to provide training for such items in university hospitals and other major tertiary hospitals. Home health care and systems and end-of-life medical treatment and care were other items that left a strong impression on trainees. At major hospitals, training tends to focus on acute care, while training on chronic diseases and palliative care is limited. Home health care and end-of-life medical treatment are primarily associated with chronic illness and palliative care, and events related to these items seemed to have a very strong impact on residents, whereas a variety of other items did not. From their reports, it appears that multifaceted training was more satisfactory than one-sided training in community medicine. CBME provides such a comprehensive training, which will help residents develop the mindset of seeing patients holistically.

It has been reported that early post-graduate work experience in rural areas positively influences residents’ choices to work in rural areas in the future [8, 9]. However, there are no reports on what specific training practices, other than practicing primary care in rural areas, can foster an attitude of comprehensive medical care and learning that differ from university hospitals, tertiary hospitals, and so on [10, 11]. Additionally, in Japan, training in rural areas does not necessarily result in healthcare professionals practicing in these same areas [12].

The strength of our study is that we explored what kind of training is needed as a CBME. Residents in our study reported experiencing significant events outside of acute care during 4 weeks of training in rural community medicine. Typically, trainees in large hospitals are trained primarily in acute care; we believe that it is important to teach aspects other than acute care in community medicine training programs. Training in primary care, integrated community care systems, medical care focusing on the local community, home health care and systems, patient-physician relationships, and end-of-life medical treatment and care, which correlate with the most frequently reported significant event items identified in this study, are considered to be important training contexts experienced during community medical care training. We believe that such training will help residents develop a mindset of caring for patients holistically through CBME.

## Limitations

This study has several limitations. First, it focused on a community medicine training program in a small rural hospital, and the results might be different in a medium-sized rural hospital or in an urban clinic. Second, because of the four-week training period, residents’ experiences are likely to differ depending on the characteristics of each training unit (such as season or number of patients). Differences across training periods may result in different significant events for each resident. Third, this study evaluated information drawn from SEA-style reports, which capture only what each resident perceived as significant during the training program and may have captured events similar to what the resident would have experienced at a major hospital.

## Conclusion

Resident trainees associated frequently some key items, such as primary care, integrated community care systems, community, end-of-life medical treatment and care, and home health care and systems with significant events. Most of these experiences can only happen in community medical care settings. Therefore, community medicine training programs should emphasize these aspects of healthcare to compensate for the limitations of training in large hospitals and clinics.

## Data Availability

The datasets used and/or analyzed during the current study are available from the corresponding author on reasonable request.

## Abbreviations

SEA: Significant event analysis
